# Stress Resistance Traits under Different Thermal Conditions in *Drosophila subobscura* from Two Altitudes

**DOI:** 10.3390/insects13020138

**Published:** 2022-01-28

**Authors:** Katarina Erić, Aleksandra Patenković, Pavle Erić, Slobodan Davidović, Marija Savić Veselinović, Marina Stamenković-Radak, Marija Tanasković

**Affiliations:** 1 Department of Genetics of Populations and Ecogenotoxicology, Institute for Biological Research “Siniša Stanković”—National Institute of the Republic of Serbia, University of Belgrade, Despot Stefan Blvd. 142, 11060 Belgrade, Serbia; aleksandra@ibiss.bg.ac.rs (A.P.); pavle.eric@ibiss.bg.ac.rs (P.E.); slobodan.davidovic@ibiss.bg.ac.rs (S.D.); marina@bio.bg.ac.rs (M.S.-R.); marija.tanaskovic@ibiss.bg.ac.rs (M.T.); 2 Faculty of Biology, University of Belgrade, Studentski Trg 3, 11000 Belgrade, Serbia; marijas@bio.bg.ac.rs

**Keywords:** *D. subobscura*, desiccation resistance, starvation resistance, chill coma recovery time, heat knock-down resistance, global warming, life history, adaptation, laboratory evolution

## Abstract

**Simple Summary:**

The global warming and rapid climate change that we are witnessing is generally influencing all of the living world, so all species must necessarily cope with these changes in order to survive. The ability to withstand environmental stress, especially during the last two decades, has been of great importance for any species’ long-term survival. For that purpose, we studied these abilities in the *Drosophila subobscura* species, which is known to be a good model organism for studying adaptations to environmental changes such as in temperature. We chose to investigate thermal stress responses in *D. subobscura* populations from two different altitudes, through four traits linked to stress tolerance: desiccation resistance, heat knock-down resistance, starvation resistance, and chill coma recovery time. Correlations between the populations’ origin and these traits were found, as well as the significant influence of the laboratory thermal conditions and sex on these traits showing that males and cold-adapted populations are expected to fare much worse in a fast-changing warming environment.

**Abstract:**

Global warming and climate change are affecting many insect species in numerous ways. These species can develop diverse mechanisms as a response to variable environmental conditions. The rise in mean and extreme temperatures due to global warming and the importance of the population’s ability to adapt to temperature stress will further increase. In this study, we investigated thermal stress response, which is considered to be one of the crucial elements of population fitness and survival in fast-changing environments. The dynamics and variation of thermal stress resistance traits in *D. subobscura* flies originating from two natural populations sampled from different altitudes were analysed. Three different temperature regimes (25 °C, 19 °C, and 16 °C) were used for the F1 progeny from both localities to establish six experimental groups and investigate stress resistance traits: desiccation resistance, heat knock-down resistance, starvation resistance, and chill-coma recovery time. We detected that laboratory thermal conditions and population origin may have an effect on the analysed traits, and that sex also significantly influences stress resistance. Individuals from the lower altitude reared at higher temperatures show inferior resistance to thermal shock.

## 1. Introduction

Global warming and climate change are associated with an increase in average and extreme temperatures. There is a growing mass of evidence that temperature is one of the foremost important factors shaping the distribution and evolution of different species populations. It is also considered to be a particularly important stressor because variable thermal environments are common and may represent substantial challenges for the survival and reproduction of these populations [1,2,3,4]. Average global temperature has risen drastically since the 19th century, and there are predictions that temperature extremes will be 1.5 °C to 4.5 °C higher than pre-industrial levels by the end of this century [5]. This increase in temperature will lead to shifts in the distribution areas of numerous species and changes in species abundance [6,7,8]. All of the known species on Earth have been affected by these changes and it is of particular interest to better understand thermal adaptation, especially in geographic gradients along which climate strongly varies. Studies of altitudinal changes in phenotype and genotype can complement studies of latitudinal patterns and provide evidence of natural selection in response to climatic factors [2,9,10].

*Drosophila* species are widely used in studies of adaptation to climate change at different biological levels, with mechanisms of adaptation and response to extreme temperatures of particular interest [9,10,11,12,13]. Their widespread abundance, small body size, short life cycle, ease to manipulate and rear and limited genetic redundancy make them good model organisms for research in biology, ecology, evolution and genetics. *D. subobscura* Collin (Diptera: Drosophilidae), a Palearctic species widespread throughout Europe, may be considered as an ideal model for thermal adaptation research due to its clinally distributed inversion polymorphism that corresponds to the warmer and colder climate and previously described population-level genetic responses to various stressful conditions [14,15,16,17,18,19,20]. A recent study [21] on temperature changes in Serbia indicated that temperature is increasing at an accelerated rate and that an increase in maximum temperature, especially during summer, is more pronounced. Previous work has indicated that *D. subobscura* populations from that region respond to thermal changes in habitat with an alteration of chromosome arrangements frequencies and changes in population structure [22]. Moreover, this species allows us short-time multigenerational maintenance, precisely controlled conditions, and a better estimation of the effects under long-term environmental changes. Latitudinal and altitudinal variations at phenotypic and genotypic levels have been thoroughly studied in *Drosophila* sp. [23,24,25,26,27,28]. For example, selection at low latitudes/altitudes (or at warm temperatures in the lab) may lead to decreased body size in these species; but the decreased size might lead to reduced stress tolerance [29].

Our aim was to try to disentangle the effect of the origin of populations according to altitude from their laboratory thermal adaptations on three fitness-related traits directly linked to thermal stress tolerance—one among which is often referred to as part of the “survival mode” mechanism which helps individuals cope with a stressful environment.

The choice of ecologically relevant traits is of special importance in studies of experimental thermal adaptation. Desiccation resistance (DR), heat knock-down resistance (HKDR), and chill coma recovery time (CCRT) are considered to be reliable indicators of thermal adaptation, all of which are related to thermal stress tolerance [23,30]. Starvation resistance (SR) is also considered to be a reliable indicator of resistance to other forms of environmental stress [31,32]. Temperature plays an important role in the evolution of these characteristics; flies that evolve at lower temperatures are typically larger, have a longer developmental time and larger eggs, unlike the flies that evolve at higher temperatures whose body size is smaller, developmental time is shorter and have smaller eggs [33,34,35,36,37,38,39,40].

In nature, high temperature is correlated with desiccation resistance and there are examples of adaptive patterns in that phenotype [41]. Desiccation is a significant stress for terrestrial animals, and the successful evolution of insects in terrestrial environments can be partly ascribed to their ability to effectively tolerate desiccation [42]. Desiccation stress continues to be a primary factor shaping insect distribution and behaviour, and an inability to respond to desiccation stress may contribute to the negative consequences of climate change for some insect species and populations. Desiccation resistance-related traits are diverse and vary in function of geographic locations and environmental conditions including water availability and environmental temperature [43,44]. The example of *Drosophila birchii* (Diptera: Drosophilidae) that appears to be restricted to the rainforest fragments due to its inability to survive desiccation indicates that if the population’s capacity to respond to selection is low, the drying of that habitat is likely to lead to extinction [35]. Even the insects that inhabit the most arid environments are ecologically successful, at least partly, because of their great capacity to conserve internal water stores [43]. Previous studies of 32 *Drosophila* species also suggested that basal resistance, rather than adult hardening (resistance based on short-term treatments under more severe stress conditions [45,46]) is relatively more important in determining species’ differences in desiccation resistance and sensitivity to climate change [44].

High temperature can affect the reproduction, mating success, abundance, and distribution of species [47,48]. Today, when many environments are becoming increasingly arid and temperature extremes are much more frequent, thermotolerance is crucial for insects’ survival. Heat knock-down resistance has been suggested as an important heat adaptation trait that correlates with natural adaptation to higher temperature environments [49]. It is suggested that the knock-down trait might be a better indicator of adaptation to natural high temperature environments than the traditionally used heat shock assays [41]. In the knock-down assay, the time taken for flies to be knocked down—become unconscious under the high temperature—is measured, reflecting the environmental conditions which disable the normal functionality of individuals.

Starvation resistance is a phenotypic trait that represents the species’ ability to withstand prolonged periods of food deprivation. Starvation is the most frequent environmental stress faced by animals inhabiting environments where food availability fluctuates and is unpredictable [50]. In natural populations, reduction in food resources and their availability are very common—challenges which are faced by animals whose capacity to survive prolonged periods of starvation is linked to their diet and nutritional status. Additionally, starvation resistance is correlated with a relatively long life span, slower development, reduced egg production, and its size, larger body size, etc. [51]. It is expected that starvation resistance increases with altitude, with the feeding resources being more limited in highlands than in lowlands [52,53], but not being coupled with altitude itself but with body size that allows the storage of higher amounts of energy. Flies of the *Drosophila* genus have been widely used to study the ecology, evolution, and genetic basis of starvation resistance, and the nutritional basis of their resistance to starvation was correlated with dietary composition, fat, and glycogen reserves [54,55,56,57,58]. Some data show the apparent association between starvation resistance and longevity in *D. melanogaster* [31]. In this study, we measured the time of death from starvation in *D. subobscura* flies from two different altitudes (populations) under three different temperature regimes as a measure of a more general response to stress conditions.

The chill coma recovery time (CCRT) describes species’ thermal adaptation and can reflect the cold tolerance of the species [31,59,60]. Cold resistance in insects has been measured in terms of survival after long-term induced stress, but short essays such as CCRT are being increasingly used. The time that ectotherms need to recover from cold stress is a nonlethal and useful index of cold tolerance [7]. CCRT is relevant for insects’ ecology and can explain variation among individuals, species, or populations. For example, in temperate climatic zones, there are daily and seasonal temperature cycles and being cold tolerant is a physical advantage during cold seasons. Freezing conditions induce coma in fruit flies and CCRT is the measure of their cold tolerance. Species such as *D. subobscura* which inhabits colder/temperate zones tend to be more chill tolerant and better cold-adapted, and they recover almost instantaneously after exposure to 0 °C, unlike tropical species [61]. To explore whether geographic populations of *D. subobscura* from different altitudes might differ in terms of their cold tolerance, we exposed them to 0 °C for some time and measured the time taken for them to stand up.

Phenotype is not a sum of different traits; rather it is a complex network of interactions of different traits and the environment surrounding it. All described separately measured fitness characteristics are good indicators of a response to a changing environment, but when analysed together, they may provide a better insight into population response to different types of thermal stress. To investigate the influence of altitude and different temperature conditions on populations’ ability to cope with temperature stress, we analysed four stress resistance traits in *D. subobscura* flies originating from two natural populations sampled from different altitudes and then maintained in three temperature regimes in laboratory conditions. The chosen temperature regimes are in range with the natural thermal experience of *D. subobscura* and are chosen as a non-lethal but stressful environment according to previously published data on thermal limitation for this species (reviewed in [62]). Past studies have indicated that laboratory maintenance at 25 °C may induce male sterility [62], 19 °C is considered to be the optimal temperature for this species [62,63] and previous but unpublished work from our laboratory suggested that 16 °C significantly prolongs development time without decreasing viability. Although limited to two populations, the results of this study add to the body of knowledge on the thermal adaptation of individuals from different latitudes. In order to predict the future abundance and distribution of species [64] and the impact of global warming on biological systems, more studies contributing to a better understanding of the mechanisms behind thermal adaptations are needed [64].

## 2. Material and Methods

Flies were collected in mid-August 2018, from two different elevations, 1080 m (N 43.395255; E 22.603995) and 1580 m (N 43.374145; E 22.618110), along the Stara planina mountain slope in Serbia. GPS was used to determine the geographical coordinates. The sampling locations were separated by an approximately 1200 m-high mountain ridge. However, as expected due to the difference in altitude, they differed in some vegetation characteristics of the habitat, both representing wild forests in closed canopy woodland. The lower locality is mainly composed beech old forest, while the higher locality is a mixture of beech and spruce. Since specific climate data could not be obtained for the sampling sites, the average temperature and humidity (H%) for both localities were measured during collection. The average temperature/humidity in the lower locality was 19.4 °C/65%, Tmax/Hmax was 28.8 °C/87.5%, and Tmin/Hmin was 13.4 °C/42.7%. In the higher locality, the average temperature/humidity was 18 °C/71%, Tmax/Hmax was 27 °C/91.2%, and Tmin/Hmin 13.5 °C/50%. As can be seen, the lower locality has a higher average and maximal temperature compared to the higher one.

The flies were collected in the evening peak of activity, by net sweeping over fermented fruit traps. Wild-caught females were individually placed into the falcon tubes to establish isofemale lines (IF). Approximately 100 flies per population were used for establishing IF lines. All lines were maintained under constant laboratory conditions: temperature of 19 °C, approximately 60% relative humidity, light of 300 lux, and 12/12 h light/dark cycles. After one generation, three to five pairs of males and females from each IF line were used to establish two synthetic mass populations (H—high altitude and L—lower altitude). Mass populations from both localities were used to establish experimental groups at three different temperature regimes: 25 °C, 19 °C, and 16 °C. All groups were maintained on a discrete generation and reared on standard *Drosophila* medium: 14 g agar, 208 g cornmeal, 188 g sugar, 40 g dry active yeast, 5 g Nipagin diluted in 60 mL of 96% ethanol in 2.2 L distilled water. Mass populations were maintained under described laboratory conditions for 12 generations controlling for larval density by transferring approximately 50 pairs to 12 food vials containing 40 mL of medium and allowing them to lay eggs for three days. After three days, parental generation was transferred to additional 12 fresh food vials for another three days and before being discarded. All the emerged progeny flies were collected, mixed, and then randomly transferred to fresh food vials as parents of the next generation. Therefore, the estimated population census counts approximately 1200 flies per generation.

After 12 generations of maintaining two populations at different temperatures under laboratory conditions, freshly emerged flies (previously kept in the dark to prevent possible mating) were collected. Males and females were separated to prevent mating and transferred to fresh food vials. All experiments, with minor alterations, followed the protocols of [52,65] and were conducted with five- to six-day-old virgin flies (Supplementary Scheme S1).

### 2.1. Desiccation Resistance (DR)

Single virgin flies were transferred into empty 3 mL tubes. Approximately 100 flies (50 ♀ and 50 ♂) were used from each group. All groups were tested for DR at three temperatures: 25 °C, 19 °C, and 16 °C. Mortality was scored every hour. Flies that were not able to move were considered dead.

### 2.2. Heath Knock-Down Resistance (HKDR)

To score HKDR, five virgin flies were placed into empty falcon tubes (50 mL). Fifty flies per group were observed. Falcon tubes were closed with moistened plugs to prevent desiccation. All groups with 50 ♀ and 50 ♂ flies per group were placed at 37 °C for seven hours, and mortality was checked every 30 min.

### 2.3. Starvation Resistance (SR)

Five virgin flies of the same sex were transferred into empty falcon tubes (50 mL) with moistened cotton plugs to prevent desiccation. Ten falcon tubes per experimental group (50 ♀ and 50 ♂) were used and placed at three temperatures: 25 °C, 19 °C, and 16 °C. Mortality was scored every three hours. Flies that were not able to move were considered dead.

### 2.4. Chill Coma Recovery Time (CCRT)

Single virgin flies were put into 3 mL tubes which are placed in a water/ice mixture. Approximately 80 (40 ♀ and 40 ♂) flies per group were used. After seven hours on ice, the tubes were moved to room temperature and the recovery time was scored for each fly (in seconds). The flies were considered to have recovered when they were able to stand up.

### 2.5. Statistics

The results for all traits were analysed using the full factorial general linear model (GLM) procedure and Bonferroni post hoc test in the STATISTICA version 12 (StatSoft. Inc. Tulsa, OK, USA). Desiccation and starvation resistance were analysed with fixed factors: population, rearing temperature, treatment, and sex, and the recovery time and heat knock-down time fixed factors for the chill coma were: population, rearing temperature, and sex.

## 3. Results

### 3.1. Desiccation Resistance

The mean values, standard error, and variance for desiccation resistance are shown in Table 1. As expected, flies died faster from desiccation under high temperature treatment conditions in all scored groups. Additionally, the females showed a higher overall desiccation resistance, especially females from a lower altitude reared at 25 °C.

**Figure 1:**
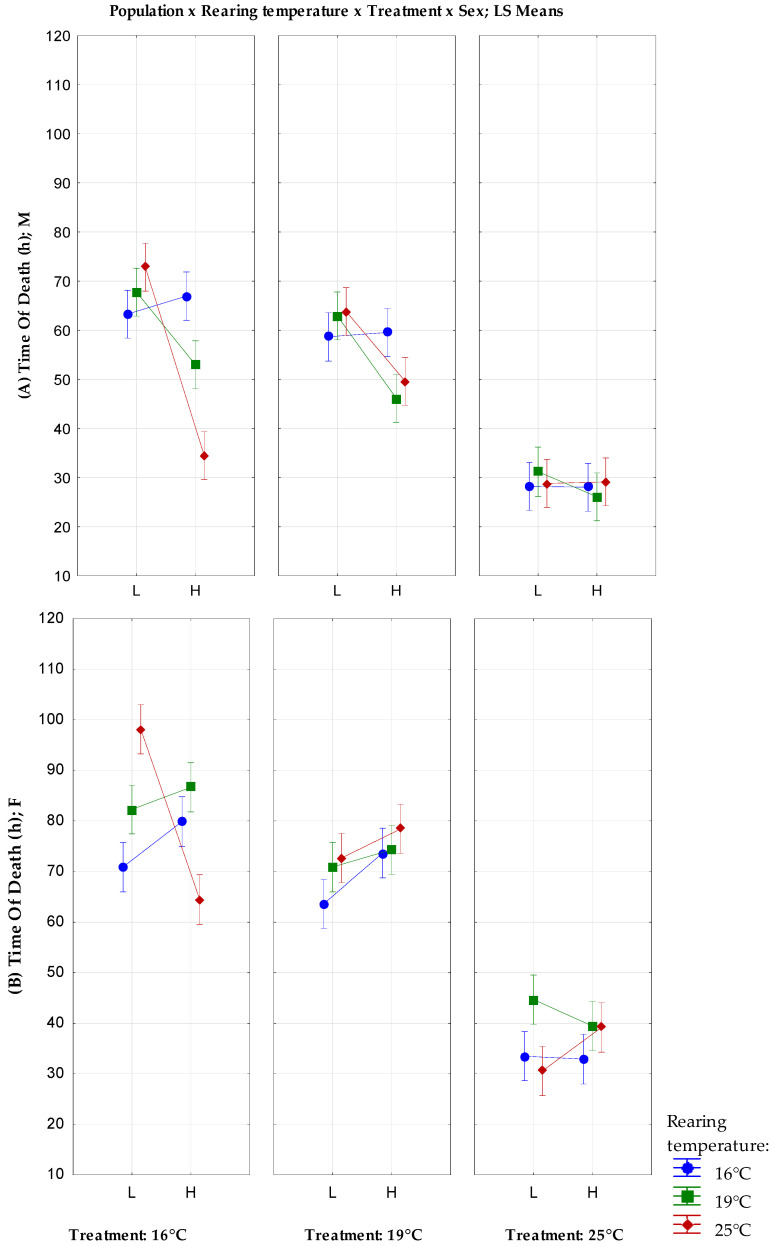
Full factorial GLM analysis of desiccation resistance with a fixed factors population, rearing temperature, treatment temperature, and sex; (**A**) males; and (**B**) females. Vertical bars denote a 0.95 confidence interval. M—males; F—females; L—population originating from lower altitude; H—population originating from the higher altitude. The modified figure was previously published in the Proceedings of the 1st International Electronic Conference on Entomology, 1–15 July 2021, doi:10.3390/IECE-10532.

The results of the full factorial GLM analysis with a fixed factors’ population, rearing temperature, treatment, and sex are shown in Figure 1. The population, treatment, and sex showed a significant influence on desiccation resistance (F_(1)_ = 30.76, *p* < 0.001; F_(2)_ = 788.14, *p* < 0.001; F_(1)_ = 315.50, *p* < 0.001, respectively (Table S1)). The rearing temperature showed no significant effect on the desiccation resistance (F_(2)_ = 2.8, *p* = 0.063 (Table S1)). There is also a significant interaction between almost all combinations of factors, except the interaction between all four factors and the population, rearing temperature, and sex combined.

Under cold temperature conditions (16 °C), both sexes from L reared at 25 °C have higher desiccation resistance than individuals from the H population, as can be seen in Figure 1 (Bonferroni *p* < 0.01 (Table S2)). Under the same conditions, L males reared at 19 °C have a higher desiccation resistance than H males (Bonferroni *p* = 0.017; Figure 1; Table S2). For females, a different although not statistically significant trend was observed, notably that the females from the H population reared at 19 °C and 16 °C showed a higher desiccation resistance than L. Under the optimal temperature for *D. subobscura* species (treatment 19 °C), L males reared at 19 °C and 25 °C had higher desiccation resistance than H males (Bonferroni *p* = 0.017, *p* = 0.035, respectively; Figure 1; Table S2). On the contrary, under the same conditions, H females had a better but not statistically significant desiccation response. Under high temperature (treatment 25 °C), there is no significant difference between groups, but H females reared at 25 °C have a higher desiccation resistance than L females.

There was no difference between males and females when flies were reared at the optimal temperature (19 °C), irrespective of population origin and the temperature of the treatment.

### 3.2. Heat Knock-Down Resistance

**Figure 2:**
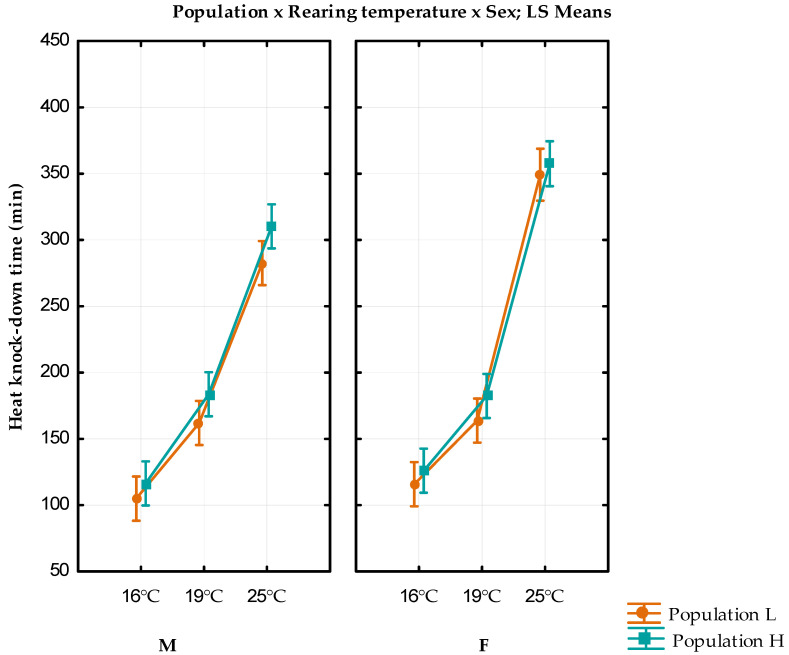
Results of full factorial GLM analysis of heat knock-down resistance with fixed factors population, rearing temperature, and sex. Vertical bars denote 0.95 confidence interval. M—males; F—females; L—population originating from lower altitude; H—population originating from a higher altitude. This Figure was previously published in Proceedings of the 1st International Electronic Conference on Entomology, 1–15 July 2021, MDPI: Basel, Switzerland, doi:10.3390/IECE-10532.

The mean values, standard errors, and variance for the heat knock-down time are given in Table 2. Individuals reared at 25 °C showed the longest heat knock-down time. The results of the full factorial GLM analysis with fixed factors population, rearing temperature, and sex are shown in Figure 2.

The population, rearing temperature, sex, and rearing temperature x sex interaction showed a statistically significant influence on the heat knock-down time (F_(1)_ = 10.707, *p* = 0.001; F_(1)_ = 20.389, *p* < 0.01, F_(2)_ = 610.236, *p* < 0.01; F_(2)_ = 11.918, *p* < 0.01, respectively (Table S3)).

Individuals from the higher altitude showed significantly longer heat knock-down time than individuals from the lower altitude (Bonferroni *p* < 0.01; Table S4). The higher rearing temperature significantly prolongs the heat knock-down time (Bonferroni *p* < 0.01 for all combinations; Table S4). For both populations, females reared at 25 °C have a significantly longer heat knock-down time than males (Bonferroni *p* = 0.006 and *p* < 0.01, respectively (Table S4)).

### 3.3. Starvation Resistance

**Figure 3:**
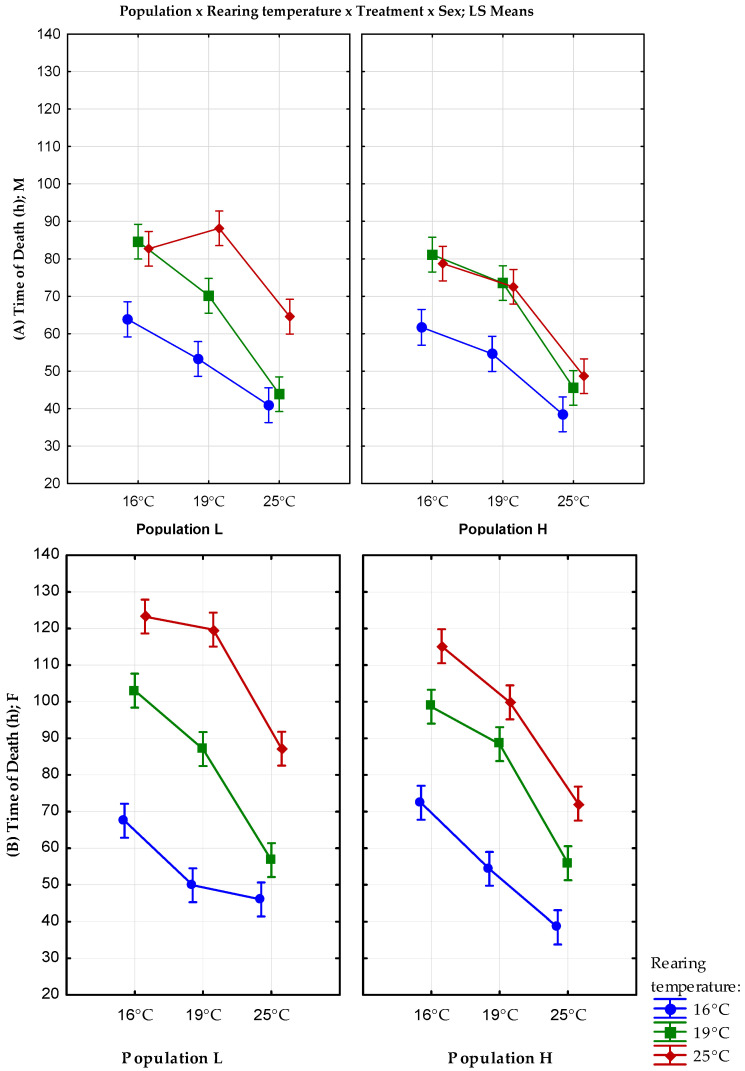
Results of the full factorial GLM analysis of starvation resistance with fixed factors population, rearing temperature, treatment temperature, and sex; (**A**) males; and (**B**) females. Vertical bars denote a 0.95 confidence interval. M—males; F—females; L—population originating from lower altitude; H—population originating from a higher altitude.

The mean values, standard errors, and variance for starvation resistance are shown in Table 3. The results of full factorial GLM analysis with fixed factors origin, rearing temperature, treatment, and sex are shown in Figure 3.

Population, rearing temperature, treatment temperature, sex, population x rearing temperature, and sex x rearing temperature interactions showed a statistically significant influence on starvation resistance (F_(1)_ = 19.4, *p* < 0.001; F_(2)_ = 356.8, *p* < 0.001, F_(2)_ = 607.57, *p* < 0.001; F_(1)_ = 238.4, *p* < 0.001; F_(2)_ = 16.6, *p* < 0.001; F_(2)_ = 58.3, *p* < 0.001, respectively (Table S5)).

Individuals reared at 25 °C showed significantly longer starvation resistance than individuals from the lower rearing temperatures. Lower altitude (L) population reared at 25 °C shows better starvation resistance under all treatments. A higher rearing temperature significantly prolongs starvation resistance, but at 25 °C, the time of death from starvation is much shorter than at lower temperatures. The females reared at 25 °C from both populations are significantly more resistant to starvation than males (Table S6).

### 3.4. Chill Coma Recovery Time

The mean values, standard errors, and variance for the chill coma recovery time are shown in Table 4. Individuals reared at 25 °C showed the longest chill coma recovery time, with significantly different values between females from both populations.

The rearing temperature and population x rearing temperature x sex interaction showed a statistically significant influence on chill coma recovery time (F_(2)_ = 111.4978, *p* < 0.001; F_(2)_ = 10.0838, *p* < 0.001, respectively; Table S7). Female individuals from a higher altitude (H) showed a significantly longer chill coma recovery time than females from lower (L) altitude (Bonferroni *p* = 0.018; Table S8); but in males, the inverted trend was observed. A higher rearing temperature significantly prolongs chill coma recovery time (Bonferroni *p* < 0.01 for all combinations). For the L population, females reared at 25 °C have a significantly shorter chill coma recovery time than their male counterparts (Bonferroni *p* = 0.002).

**Figure 4:**
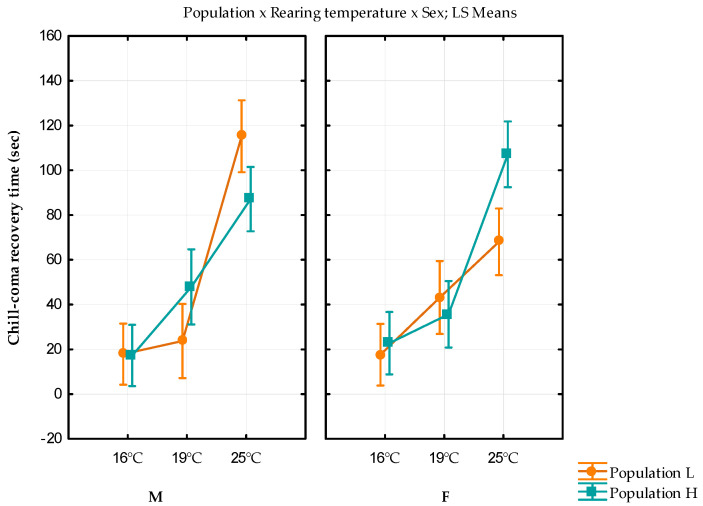
Results of the full factorial GLM analysis for chill coma recovery time with fixed factors population, rearing temperature, and sex. Vertical bars denote 0.95 confidence interval. M—males; F—females; L—population originating from lower altitude; and H—population originating from a higher altitude.

The results of the full factorial GLM analysis with fixed factors population, rearing temperature, and sex are shown in Figure 4.

## 4. Discussion

Organisms in populations of different species are coping with the impacts of climate change and consequently with environmental changes that occur very rapidly, so they must adapt to these changes in order to survive. Extreme temperatures, periods of droughts, or food deficiency which are happening in every environment can wipe out a population unless it is capable of adapt to those conditions or escape in some other way [66]. In any given species, the shape and the speed of adaptation depend on numerous factors, such as the studied trait, population sampled, environmental conditions, and type of response [64]. Temperature is one of the most important selective agents, and studies of laboratory thermal evolution may provide insight into adaptation mechanisms as the responses to climatic change [35]. The present study analyses the dynamics and variation of four stress resistance traits, namely desiccation resistance, heat knock-down resistance, starvation resistance, and chill coma recovery time in *D. subobscura* flies originating from natural populations from two different altitudes kept under different strictly defined temperature regimes under laboratory conditions.

Our results suggest that population origin has a significant influence on all analysed traits, except CCRT. For desiccation resistance, the lower altitude population generally responds better to desiccation than the high-altitude population, which is expected considering the fact that high temperature is a cause of desiccation in the wild, and temperature generally decreases with increasing altitude [23]. For heat knock-down resistance, we have a different population response, where the high-altitude population showed better resistance to heat knock-down. At higher altitudes, weather conditions are generally correlated with temperature extremes, and population H reared at 25 °C showed the highest resistance to short-term extreme temperature. The results of the starvation resistance assay showed that the L population is more tolerant to food shortages than the H population. The flies reared at 16 °C were the least starvation tolerant, and flies reared at 25 °C were the most resistant to food deprivation. This can be explained by temperature acclimation and the possibility that highland populations do not have the ability to cope with the deficiency of food and very high temperature at the same time. A recovery time from chill coma takes the longest time for flies reared at 25 °C, while the flies reared at 16 °C recovered almost instantly, which could be explained with their preacclimation to a certain temperature. We did not notice a significant difference between the populations’ responses to CCRT, but this result is not surprising as differences in this trait were not observed in *Drosophila melanogaster* populations from distinct geographic and climate origins [67]. The chill tolerance traits are thus mediated by different physiological mechanisms and measuring more than one trait is important for evidence of temperature selection pressure. The influence of population origin, detected for three of the four analysed traits, is an indication that altitude may have an influence on shaping the response to temperature stress. However, deciphering the influence of altitude population distribution on thermal stress traits may require the analysis of several independent low- and high-altitude populations to further corroborate these claims. Considering the fact that only two populations from different altitudes are included in our analysis, we cannot exclude some other mechanisms such as drift which play a significant role in shaping population response to thermal stress—as detected in our study. However, even though this species is known for its high mobility and mostly large effective population size, local adaptations at a small spatial scale have been described [68]. Moreover, local adaptation to colder and warmer environments for reproductive performance traits in two *D. subobscura* populations was described [69], indicating that fitness traits depend on both the thermal origin and laboratory conditions of the population and that, if local conditions are different, two populations may be sufficient to support such claims.

Our results suggest that laboratory thermal conditions have an effect on all analysed traits, but as expected, this is more prominent for HKDR and CCRT, which are positively correlated with a high developmental temperature, as previously shown in *D. melanogaster* and *D. buzzati* [31,41,42,67]. As previously shown, adaptation to a colder environment decreases HKDR [70] and our results suggest that with the increased rearing temperature, HKDR and CCRT are prolonged, indicating a trade-off between HKDR and CCRT at a high rearing temperature. Previously published work in *D. subobscura* showed that as few as nine generations of laboratory thermal conditions may lead to the rapid and consistent evolution of wing shape [71], indicating that adaptation to laboratory thermal conditions may take a relatively short time period. Although the lab adaptation process is faster in the first 15–20 generations, it still extends to many more generations after [72]. Our results suggest that the thermal regime that individuals experienced in the laboratory for 12 generations influence the population’s ability to endure temperature extremes, indicating that individuals accustomed to colder environments may face serious challenges in light of global warming. Our results suggest that laboratory thermal conditions and adaptations to imposed thermal conditions influence the population response to desiccation and starvation resistance under short-term cold conditions, but do not have the same influence under higher temperature conditions. Although our analysis detected the influence of laboratory thermal conditions on all analysed traits, we cannot exclude the effect of developmental plasticity as a factor contributing to the observed patterns and shaping individuals response. Our results add to the body of knowledge that temperature extremes, whether high or low, may have a stronger effect on populations preadapted to lower temperatures, and extreme fluctuations may jeopardise their ability to adapt and survive [40,65]. Further studies on more populations of different geographical origin and following an experimental evolution design including replication are needed to better understand the true effect of extreme temperature fluctuation on population ability to survive and adapt to such conditions.

Not surprisingly, all the traits apart from CCRT were strongly influenced by sex. Females, as we previously described, showed better heat resistance performances than males, indicating that males have more difficulties coping with rising temperatures. Females have overall better DR, SR, and HKDR. A previous work showed that an elevated temperature has diverse effects on the different sexes of this species and that, for instance, exposure to 25 °C can even induce male sterility in *D. subobscura* [62]. Additionally, it has been suggested that mating success under heat stress in *D. buzzati* can be a direct target of thermal selection and that thermal sexual selection has a negative effect on cold resistance [49]. We noted that with an elevated rearing temperature, the CCRT for females from the L population needed a shorter time to recover than males, but in the H population, a different trend was observed, possibly indicating preadaptation to harsher environmental conditions.

*Drosophila suboscura* is considered a cold-adapted species with a thermal range between 6 °C and 26 °C [73] and a thermal optimum of 18 °C [62]. Behavioural assays revealed that this species shows a thermal preference of 16.6 °C when placed in a linear thermal gradient [63]. Desiccation, heat knock-down resistance, and chill coma recovery are considered reliable indicators of thermal adaptation, all of which suggest that these correlate with natural adaptation to high-temperature environments [35]. Although the temperatures used in this experiment are not extreme, in the range experienced by *D. subobscura* developing in nature, our results show that almost all traits are sensitive to rearing temperature and to the origin of the population. However, the patterns of the traits did not respond the same: they show different sensitivities to non-optimal temperatures, indicating different mechanisms responsible for thermal stress adaptation. For instance, for HKDR and CCRT, flies have different responses depending on their rearing temperature, which was expected. The population from a high altitude (H) showed better HKDR and CCRT which can be correlated with a better tolerance to extreme weather conditions in the highlands. The patterns of knock-down resistance and chill coma recovery correlate well with the thermal history of the two populations investigated, where flies reared at 25 °C were shown to be the most heat tolerant via an HKDR assay, but also the less cold tolerant via a CCRT assay, which is indicative of a trade-off association between this two traits regarding rearing temperature. For flies reared at 16 °C, the opposite results were obtained. The lowland L population showed an overall better resistance to desiccation and starvation resistance. Higher rearing temperature prolongs survival for both desiccation and starvation, suggesting that preadaptation to warmer conditions plays an important role in the mechanisms enabling the organism to cope with different environments. Desiccation is significant stress for terrestrial animals, specifically for insects, which are particularly vulnerable to the loss of water due to their relatively small body size [43]. Our results indicate that flies reared at 16 °C and tested for desiccation resistance at 25 °C are the least desiccation tolerant of all groups, especially the highland population, probably because this population is preadapted to cold environment.

There is clear evidence that many habitats are becoming increasingly threatened by stressful climate changes, and anthropogenic factors are altering thermal conditions and are also mostly responsible for the increased rates of current and expected future extinctions. To understand the effects of climate warming on some species and ecosystems, long-term observations of the occurrence of species and detailed knowledge on their ecology and life-history is crucial, but studies such as this one, despite its limitations, can also make a significant contribution to understanding how species will respond to ongoing climate change. Environmental stress resistance traits are complex quantitative genetic traits that are influenced by the combined effect of genes and environmental conditions. Our results suggest that both long- (rearing temperature) and short-term (HKDR and CCRT temperatures) exposure to a high temperature has a greater detrimental influence on all analysed traits compared to any low temperature exposure. Population history is also an important factor that shapes the individual response to suboptimal and extreme temperature stress, but again, heat stress has a more pronounced effect than cold stress. In light of global warming, our results add to the body of knowledge that cold-adapted species are expected to fare much worse in a fast-changing environment.

## Figures and Tables

**Table 1:** Mean values with standard errors and variance for all groups for a time of death (measured in hours) under desiccation stress. Population L—population originating from lower altitude; population H—population originating from higher altitude; rearing temperature—T at which flies evolve in the laboratory; treatment—T at which flies were tested for desiccation resistance; F—females; M—males; time of death—mean value ± SE (standard error). This Table was published in the *Proceedings of the 1st International Electronic Conference on Entomology*, 1–15 July 2021, doi:10.3390/IECE-10532.

			Population L		Population H	
Rearing Temperature	Treatment	Sex	Time of Death	Variance	Time of Death	Variance
16 °C	16 °C	F	70.86 ± 2.06	214.1229	79.85 ± 2.84	394.25
		M	63.28 ± 1.83	167.3486	66.98 ± 2.76	373.8954
	19 °C	F	63.6 ± 2.35	134.8163	73.63 ± 2.35	269.6539
		M	58.67 ± 1.91	178.4328	59.54 ± 1.42	67.84354
	25 °C	F	33.48 ± 1.44	103.6016	32.90 ± 1.18	100.2943
		M	28.2 ± 0.91	41.71429	28.04 ± 0.93	43.26367
19 °C	16 °C	F	82.23 ± 3.16	510.4235	86.64 ± 3.10	481.8678
		M	67.76 ± 2.22	246.9616	52.98 ± 2.33	249.5302
	19 °C	F	70.86 ± 2.37	280.6535	74.28 ± 1.66	138.5322
		M	62.94 ± 2.17	234.5065	46.14 ± 1.42	100.7759
	25 °C	F	44.68 ± 1.19	159.5282	39.44 ± 1.35	90.45551
		M	30.28 ± 1.02	52.36898	26.1 ± 1.03	53.43878
25 °C	16 °C	F	98.14 ± 5.00	1250.735	64.44 ± 4.06	823.1902
		M	72.84 ± 4.21	886.7086	34.46 ± 2.18	237.2739
	19 °C	F	72.64 ± 5.01	1256.235	78.4 ± 4.18	872.0816
		M	63.82 ± 2.67	356.6404	49.62 ± 2.22	245.5873
	25 °C	F	30.56 ± 1.42	100.8229	39.16 ± 1.91	181.6065
		M	28.82 ± 1.58	125.0486	29.14 ± 1.17	68.57184

**Table 2:** Mean values with standard errors and variance for all groups for the heat knock-down time (measured in minutes). Population L—population originating from lower altitude; population H—population originating from higher altitude; rearing temperature—T at which flies evolve in the laboratory; F—females; M—males; heat knock-down time—time by flies have been knocked down (mean value ± SE (standard error)). This Table was previously published in *Proceedings of the 1st International Electronic Conference on Entomology*, 1–15 July 2021, MDPI: Basel, Switzerland, doi:10.3390/IECE-10532.

		Population L		Population H	
Rearing Temperature	Sex	Heat Knock-Down Time	Variance	Heat Knock-Down Time	Variance
16 °C	F	115.8 ± 3.93	771.7959	126 ± 3.53	624.4898
	M	105 ± 2.74	376.5306	116.4 ± 3.16	501.0612
19 °C	F	163.8 ± 3.66	668.9388	182.4 ± 5.06	1279.837
	M	162 ± 3.64	661.2245	183.6 ± 4.67	1088.816
25 °C	F	310.2 ± 12.99	8434.653	357.5 ± 14.06	9491.489
	M	349.17 ± 17.77	11,373.57	282.6 ± 14.32	10,252.29

**Table 3:** Mean values with standard errors and variance for all groups for starvation resistance (measured in hours). Population L—population originating from lower altitude; population H—population originating from higher altitude; rearing temperature—T at which flies evolve in the laboratory; treatment—T at which flies were tested for starvation resistance; F—females; M—males; time of death—mean value ± SE (standard error).

			Population L		Population H	
Rearing Temperature	Treatment	Sex	Time of Death	Variance	Time of Death	Variance
16 °C	16 °C	F	67.5 ± 2.26	256.1327	72.42 ± 2.45	300.8608
		M	63.86 ± 1.58	121.875	61.72 ± 2.05	197.1175
	19 °C	F	48.88 ± 2.10	221.2914	54.36 ± 1.71	147.1739
		M	53.58 ± 1.30	85.79755	55.75 ± 1.79	154.2766
	25 °C	F	46.02 ± 0.99	48.71388	38.46 ± 1.35	90.62082
		M	40.92 ± 0.70	24.52408	38.46 ± 0.93	43.23306
19 °C	16 °C	F	103.02 ± 3.42	585.0404	98.64 ± 3.02	455.0106
		M	84.6 ± 1.92	184.0408	81.12 ± 1.81	161.9853
	19 °C	F	87.06 ± 1.90	180.9147	88.44 ± 2.02	204.7004
		M	70.14 ± 2.29	262.6127	73.5 ± 2.18	237.398
	25 °C	F	56.76 ± 1.54	117.8596	55.92 ± 1.48	109.3812
		M	43.86 ± 1.00	50.28612	45.54 ± 0.92	42.86571
25 °C	16 °C	F	123.24 ± 3.48	604.5943	115.4 ± 2.61	341.5922
		M	82.68 ± 2.41	290.6302	78.72 ± 2.80	391.4302
	19 °C	F	119.7 ± 3.19	508.1327	99.84 ± 3.98	790.5453
		M	88.14 ± 2.03	207.1433	72.54 ± 3.44	593.1514
	25 °C	F	87.18 ± 3.20	512.2322	72.18 ± 4.12	851.6608
		M	64.56 ± 1.99	197.7208	48.66 ± 2.82	396.4739

**Table 4:** Mean values with standard errors and variance for all groups for chill-coma recovery time (measured in minutes). Population L—population originating from lower altitude; population H—population originating from higher altitude; rearing temperature—T at which flies evolve in the laboratory; F—females; M—males; chill coma recovery time—time by flies were recovered from CCRT (mean value ± SE (standard error)).

		Population L		Population H	
Rearing Temperature	Sex	Chill Coma Recovery Time	Variance	Chill Coma Recovery Time	Variance
16 °C	F	17.61 ± 1.72	143.7924	22.74 ± 1.75	146.3962
	M	17.85 ± 1.77	156.0721	17.26 ± 1.83	167.116
19 °C	F	43.14 ± 5.76	1160.334	35.65 ± 2.53	268.8758
	M	23.72 ± 2.46	206.3637	65.80 ± 12.67	6901.601
25 °C	F	68.04 ± 9.10	3481.358	107.11 ± 14.31	8810.542
	M	115.21 ± 15.60	8751.539	87.09 ± 9.99	4484.564

## Data Availability

The data presented in this study are available in article.

## Supplementary Materials

The following are available online at https://www.mdpi.com/article/10.3390/insects13020138/s1, Table S1: Results of GLM for desiccation resistance; Table S2: Bonferroni correction for all pairwise comparisons for desiccation resistance; Table S3: Results of GLM for heat knock-down resistance; Table S4: Bonferroni correction for all pairwise comparisons for heat knock-down resistance; Table S5: Results of GLM for starvation resistance; Table S6: Bonferroni correction for all pairwise comparisons for starvation resistance; Table S7: Results of GLM for chill coma recovery time; Table S8: Bonferroni correction for all pairwise comparisons for chill coma recovery time; Supplementary Scheme S1: Experimental scheme.

## References

[1] Fischer K., Dierks A., Franke K., Geister T.L., Liszka M., Winter S., Pflicke C. (2010). Environmental Effects on Temperature Stress Resistance in the Tropical Butterfly Bicyclus Anynana. PLoS ONE.

[2] Karl I., Janowitz S.A., Fischer K. (2008). Altitudinal life-history variation and thermal adaptation in the copper butterfly *Lycaena tityrus*. Oikos.

[3] Walsh B.S., Parratt S.R., Hoffmann A.A., Atkinson D., Snook R.R., Bretman A., Price T.A.R. (2019). The Impact of Climate Change on Fertility. Trends Ecol. Evol..

[4] Buckley L.B., Kingsolver J.G. (2021). Evolution of Thermal Sensitivity in Changing and Variable Climates. Annu. Rev. Ecol. Evol. Syst..

[5] IPCC (2018). The Intergovernmental Panel on Climate Change.

[6] Kumar N.H., Murali M., Girish H., Chandrashekar S., Amruthesh K., Sreenivasa M., Jagannath S. (2021). Impact of climate change on biodiversity and shift in major biomes. Global Climate Change.

[7] David J.R., Gibert P., Moreteau B., Gilchrist G.W., Huey R.B. (2003). The Fly That Came in from the Cold: Geographic Variation of Recovery Time from Low-Temperature Exposure in *Drosophila subobscura*. Funct. Ecol..

[8] Warren R., Price J., Jenkins R., Letcher T.M. (2021). Chapter 4—Climate change and terrestrial biodiversity. The Impacts of Climate Change.

[9] Markow T.A. (2015). The secret lives of *Drosophila* flies. eLife.

[10] Balanyà J., Huey R.B., Gilchrist G.W., Serra L. (2009). The chromosomal polymorphism of *Drosophila subobscura*: A microevolutionary weapon to monitor global change. Heredity.

[11] Loeschcke V., Sørensen J.G., Kristensen T.N. (2004). Ecologically relevant stress resistance: From microarrays and quantitative trait loci to candidate genes—A research plan and preliminary results using *Drosophila* as a model organism and climatic and genetic stress as model stresses. J. Biosci..

[12] Kristensen T.N., Dahlgaard J., Loeschcke V. (2003). Effects of inbreeding and environmental stress on fitness—Using *Drosophila buzzatii* as a model organism. Conserv. Genet..

[13] Xue Q., Majeed M.Z., Zhang W., Ma C.-S. (2019). Adaptation of Drosophila species to climate change—A literature review since 2003. J. Integr. Agric..

[14] Collinge J.E., Hoffmann A.A., McKechnie S.W. (2006). Altitudinal patterns for latitudinally varying traits and polymorphic markers in *Drosophila melanogaster* from eastern Australia. J. Evol. Biol..

[15] Kenig B., Jelić M., Kurbalija Z., Stamenković-Radak M., Anđelković M. (2010). Inversion polymorphism in populations of *Drosophila subobscura* from urban and non-urban environment. Arch. Biol. Sci..

[16] Rodríguez-Trelles F., Rodríguez M.A. (1998). Rapid micro-evolution and loss of chromosomal diversity in *Drosophila* in response to climate warming. Evol. Ecol..

[17] Rodríguez–Trelles F., Rodríguez M.A., Scheiner S.M. (1998). Tracking the genetic effects of global warming: *Drosophila* and other model systems. Conserv. Ecol..

[18] Davis A.J., Lawton J.H., Shorrocks B., Jenkinson L.S. (1998). Individualistic species responses invalidate simple physiological models of community dynamics under global environmental change. J. Anim. Ecol..

[19] Stamenkovic-Radak M., Kenig B., Djurakic M.R., Jelj M., Eric K., Andjelkovj M. (2019). Associations between environmental variability and inversion polymorphism of *Drosophila subobscura*: Meta-analysis of populations from the Central Balkans. Clim. Res..

[20] Rezende E.L., Balanyà J., Rodríguez-Trelles F., Rego C., Fragata I., Matos M., Serra L., Santos M. (2010). Climate change and chromosomal inversions in *Drosophila subobscura*. Clim. Res..

[21] Vuković A.J., Vujadinović M.P., Rendulić S.M., Đurđević V.S., Ruml M.M., Babić V.P., Popović D.P. (2018). Global warming impact on climate change in Serbia for the period 1961-2100. Therm. Sci..

[22] Stamenkovic-Radak M., Rasic G., Savic T., Kalajdzic P., Kurbalija Z., Kenig B., Andjelkovic M. (2008). Monitoring of the genetic structure of natural populations: Change of the effective population size and inversion polymorphism in *Drosophila subobscura*. Genetica.

[23] Ayhan N., Güler P., Onder B.S. (2016). Altitudinal variation in lifespan of *Drosophila melanogaster* populations from the Firtina Valley, northeastern Turkey. J. Therm. Biol..

[24] Dahlgaard J., Hasson E., Loeschcke V. (2001). Behavioral differentiation in oviposition activity in *Drosophila buzzatii* from highland and lowland populations in argentina: Plasticity or thermal adaptation?. Evol. Int. J. Org. Evol..

[25] Parkash R., Tyagi P.K., Sharma I., Rajpurohit S. (2005). Adaptations to environmental stress in altitudinal populations of two *Drosophila* species. Physiol. Entomol..

[26] Sambucetti P., Loeschcke V., Norry F.M. (2006). Developmental time and size-related traits in *Drosophila buzzatii* along an altitudinal gradient from Argentina. Hereditas.

[27] Pitchers W., Pool J.E., Dworkin I. (2013). Altitudinal clinal variation in wing size and shape in African *Drosophila melanogaster*: One cline or many?. Evol. Int. J. Org. Evol..

[28] Klepsatel P., Gáliková M., Huber C.D., Flatt T. (2014). Similarities and differences in altitudinal versus latitudinal variation for morphological traits in *Drosophila melanogaster*. Evol. Int. J. Org. Evol..

[29] Bijlsma R., Loeschcke V. (2005). Environmental stress, adaptation and evolution: An overview. J. Evol. Biol..

[30] Sgrò C.M., Overgaard J., Kristensen T.N., Mitchell K.A., Cockerell F.E., Hoffmann A.A. (2010). A comprehensive assessment of geographic variation in heat tolerance and hardening capacity in populations of *Drosophila melanogaster* from eastern Australia. J. Evol. Biol..

[31] Rion S., Kawecki T.J. (2007). Evolutionary biology of starvation resistance: What we have learned from *Drosophila*. J. Evol. Biol..

[32] Kellermann V., Hoffmann A.A., Kristensen T.N., Moghadam N.N., Loeschcke V. (2015). Experimental Evolution under Fluctuating Thermal Conditions Does Not Reproduce Patterns of Adaptive Clinal Differentiation in *Drosophila melanogaster*. Am. Nat..

[33] Erić K., Erić P., Davidović S., Veselinović M.S., Patenković A., Stamenković-Radak M., Tanasković M. Thermal Adaptation and Stress Resistance in *D. subobscura* Populations from Two Altitudes at Stara Planina Mountain (Serbia). Proceedings of the 1st International Electronic Conference on Entomology.

[34] Partridge L., Barrie B., Fowler K., French V. (1994). Evolution and development of body size and cell size in *Drosophila melanogaster* in response to temperature. Evol. Int. J. Org. Evol..

[35] Hoffmann A.A., Sørensen J.G., Loeschcke V. (2003). Adaptation of *Drosophila* to temperature extremes: Bringing together quantitative and molecular approaches. J. Therm. Biol..

[36] Zamudio K.R., Huey R.B., Crill W.D. (1995). Bigger isn’t always better: Body size, developmental and parental temperature and male territorial success in *Drosophila melanogaster*. Anim. Behav..

[37] French V., Feast M., Partridge L. (1998). Body size and cell size in *Drosophila*: The developmental response to temperature. J. Insect Physiol..

[38] McCabe J., Partridge L. (1997). An interaction between environmental temperature and genetic variation for body size for the fitness of adult female *Drosophila melanogaster*. Evol. Int. J. Org. Evol..

[39] Reeve M., Fowler K., Partridge L. (2000). Increased body size confers greater fitness at lower experimental temperature in male *Drosophila melanogaster*. J. Evol. Biol..

[40] Gilchrist G.W., Jeffers L.M., West B., Folk D.G., Suess J., Huey R.B. (2008). Clinal patterns of desiccation and starvation resistance in ancestral and invading populations of *Drosophila subobscura*. Evol. Appl..

[41] Sorensen J.G., Dahlgaard J., Loeschcke V. (2001). Genetic Variation in Thermal Tolerance among Natural Populations of *Drosophila buzzatii*: Down Regulation of Hsp70 Expression and Variation in Heat Stress Resistance Traits. Funct. Ecol..

[42] Chown S.L., Chown S., Nicolson S. (2004). Insect Physiological Ecology: Mechanisms and Patterns.

[43] Krupp J.J., Nayal K., Wong A., Millar J.G., Levine J.D. (2020). Desiccation resistance is an adaptive life-history trait dependent upon cuticular hydrocarbons, and influenced by mating status and temperature in *D. melanogaster*. J. Insect Physiol..

[44] Kellermann V., McEvey S.F., Sgrò C.M., Hoffmann A.A. (2020). Phenotypic Plasticity for Desiccation Resistance, Climate Change, and Future Species Distributions: Will Plasticity Have Much Impact?. Am. Nat..

[45] Bowler K. (2005). Acclimation, heat shock and hardening. J. Therm. Biol..

[46] Loeschcke V., Sørensen J.G. (2005). Acclimation, heat shock and hardening—A response from evolutionary biology. J. Therm. Biol..

[47] Franks S.J., Hoffmann A.A. (2012). Genetics of Climate Change Adaptation. Annu. Rev. Genet..

[48] Hoffmann A.A., Sgrò C.M. (2011). Climate change and evolutionary adaptation. Nature.

[49] Stazione L., Norry F.M., Gomez F.H., Sambucetti P. (2020). Heat knockdown resistance and chill-coma recovery as correlated responses to selection on mating success at high temperature in *Drosophila buzzatii*. Ecol. Evol..

[50] McCue M.D. (2010). Starvation physiology: Reviewing the different strategies animals use to survive a common challenge. Comp. Biochem. Physiol. Part A Mol. Integr. Physiol..

[51] Schwasinger-Schmidt T.E., Kachman S.D., Harshman L.G. (2012). Evolution of starvation resistance in *Drosophila melanogaster*: Measurement of direct and correlated responses to artificial selection. J. Evol. Biol..

[52] Sørensen J.G., Norry F.M., Scannapieco A.C., Loeschcke V. (2005). Altitudinal variation for stress resistance traits and thermal adaptation in adult *Drosophila buzzatii* from the New World. J. Evol. Biol..

[53] Hasson E., Rodríguez C., Fanara J.J., Naveira H., Reig O.A., Fontdevila A. (1995). The evolutionary history of *Drosophila buzzatti*. XXVI. Macrogeographic patterns of inversion polymorphism in New World populations. J. Evol. Biol..

[54] Lee K.P., Jang T. (2014). Exploring the nutritional basis of starvation resistance in *Drosophila melanogaster*. Funct. Ecol..

[55] Skorupa D.A., Dervisefendic A., Zwiener J., Pletcher S.D. (2008). Dietary composition specifies consumption, obesity, and lifespan in *Drosophila melanogaster*. Aging Cell.

[56] Chippindale A.K., Chu T.J., Rose M.R. (1996). Complex trade-offs and the evolution of starvation resistance in *Drosophila melanogaster*. Evol. Int. J. Org. Evol..

[57] Djawdan M., Chippindale A.K., Rose M.R., Bradley T.J. (1998). Metabolic reserves and evolved stress resistance in *Drosophila melanogaster*. Physiol. Zool..

[58] Hoffmann A.A., Harshman L.G. (1999). Desiccation and starvation resistance in *Drosophila*: Patterns of variation at the species, population and intrapopulation levels. Heredity.

[59] Hoffmann A.A., Hallas R., Anderson A.R., Telonis-Scott M. (2005). Evidence for a robust sex-specific trade-off between cold resistance and starvation resistance in *Drosophila melanogaster*. J. Evol. Biol..

[60] Andersen J.L., Manenti T., Sørensen J.G., MacMillan H.A., Loeschcke V., Overgaard J. (2015). How to assess Drosophila cold tolerance: Chill coma temperature and lower lethal temperature are the best predictors of cold distribution limits. Funct. Ecol..

[61] Gibert P., Moreteau B., Pétavy G., Karan D., David J.R. (2001). Chill-coma tolerance, a major climatic adaptation among drosophila species. Evol. Int. J. Org. Evol..

[62] Krimbas C.B., Krimpas K.V. (1993). Drosophila subobscura: Biology, Genetics and Inversion Polymorphism.

[63] Rego C., Balanyà J., Fragata I., Matos M., Rezende E.L., Santos M. (2010). Clinal patterns of chromosomal inversion polymorphisms in *Drosophila subobscura* are partly associated with thermal preferences and heat stress resistance. Evol. Int. J. Org. Evol..

[64] Kristensen T.N., Ketola T., Kronholm I. (2020). Adaptation to environmental stress at different timescales. Ann. N. Y. Acad. Sci..

[65] Hoffmann A.A., Anderson A., Hallas R. (2002). Opposing clines for high and low temperature resistance in *Drosophila melanogaster*. Ecol. Lett..

[66] Hoffmann A.A., Sgrò C.M., Kristensen T.N. (2017). Revisiting Adaptive Potential, Population Size, and Conservation. Trends Ecol. Evol..

[67] Davis H.E., Cheslock A., MacMillan H.A. (2021). Chill coma onset and recovery fail to reveal true variation in thermal performance among populations of *Drosophila melanogaster*. Sci. Rep..

[68] Savic Veselinovic M., Kurbalija Novicic Z., Kenig B., Jelic M., Patenkovic A., Tanaskovic M., Pertoldi C., Stamenkovic-Radak M., Andjelkovic M. (2019). Local adaptation at fine spatial scale through chromosomal inversions and mito-nuclear epistasis: Findings in *Drosophila subobscura* (Diptera: Drosophilidae). Eur. J. Entomol..

[69] Porcelli D., Gaston K.J., Butlin R.K., Snook R.R. (2017). Local adaptation of reproductive performance during thermal stress. J. Evol. Biol..

[70] Kimura M.T. (2004). Cold and heat tolerance of drosophilid flies with reference to their latitudinal distributions. Oecologia.

[71] Santos M., Iriarte P.F., Céspedes W., Balanyà J., Fontdevila A., Serra L. (2004). Swift laboratory thermal evolution of wing shape (but not size) in *Drosophila subobscura* and its relationship with chromosomal inversion polymorphism. J. Evol. Biol..

[72] Simões P., Fragata I., Santos J., Santos M.A., Santos M., Rose M.R., Matos M. (2019). How phenotypic convergence arises in experimental evolution. Evol. Int. J. Org. Evol..

[73] Moreteau B., Morin J.P., Gibert P., Pétavy G., Pla É., David J.R. (1997). Evolutionary changes of non linearreaction norms according to thermal adaptation: A comparison of two Drosophila species. C. R. Acad. Sci..

