# Supplementary figures and images for: Stress Resistance Traits under Different Thermal Conditions in Drosophila subobscura from Two Altitudes
Source: Insects. 2022 Jan 28;13(2):138. doi: 10.3390/insects13020138 (PMC8875991; doi:10.3390/insects13020138)

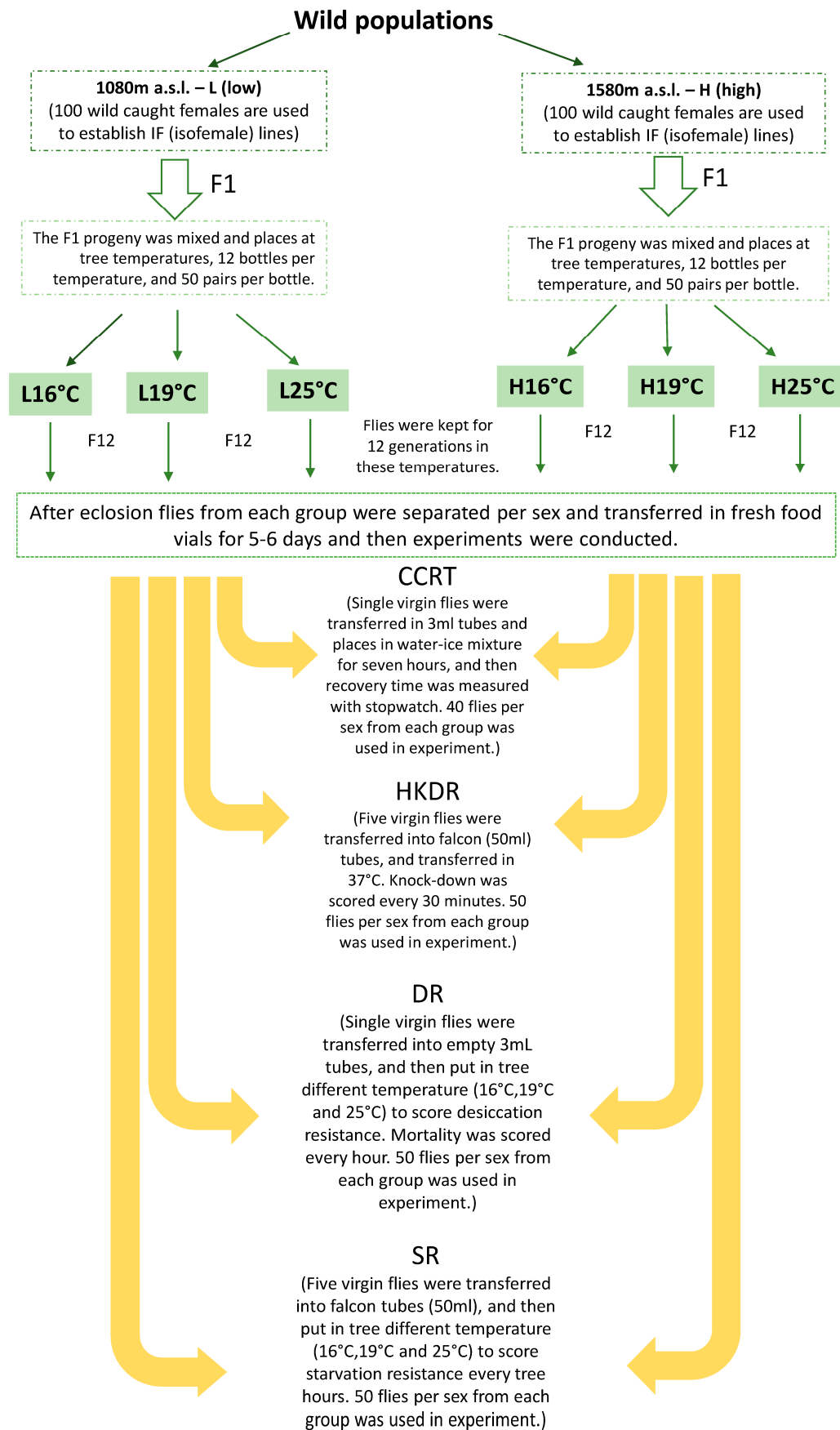

**Scheme S1.** Experimental scheme.

Supplement: Supplementary file 1 [file insects-13-00138-s001.zip › Supplementary Scheme S1.pdf]
